# Evolutionary and pan-genomic analysis of the *bZIP* gene family in 21 *Camellia sinensis*

**DOI:** 10.3389/fpls.2026.1885680

**Published:** 2026-07-03

**Authors:** Quanlong Liu, Jiaqi Lian, Hongmei Tang

**Affiliations:** 1 School of Life Sciences, North China University of Science and Technology, Tangshan, China; 2 School of Life Science and Technology, Northwestern Polytechnical University, Xi’an, Shaanxi, China

**Keywords:** *bZIP* transcription factors, *Camellia sinensis*, copy number variation, drought stress, pangenome

## Abstract

Basic leucine zipper (*bZIP*) transcription factors are important regulators of plant development and stress responses, yet their evolutionary dynamics in tea plant have largely been inferred from a single reference genome. Here, we performed a broad evolutionary and pan-genomic analysis of the *bZIP* family using 1,015 plant genomes and 21 *Camellia sinensis* genomes. Across plants, 81,340 *bZIP* genes were identified, revealing broad conservation of this family across major lineages and a significant copy-number expansion in angiosperms. In tea, 1,635 non-redundant *bZIP* genes were identified, with 73–88 members per genome, indicating an overall conserved family size among tea germplasms. Phylogenetic analysis classified these genes into 13 subfamilies, among which S, A, D, I and G represented the major expanded groups. Orthogroup analysis resolved 77 *bZIP* orthogroups, including 22 core orthogroups and 55 dispensable orthogroups, suggesting substantial hidden variation despite stable total gene numbers. WGD/segmental duplication was the dominant expansion mechanism, accounting for 59.20% of tea *bZIP* genes, followed by dispersed duplication. Copy-number variation was widespread, with 72 of 77 orthogroups showing CNV across genomes. Most homologous gene pairs evolved under purifying selection, whereas dispensable genes exhibited relatively relaxed constraints compared with core genes. Transcriptome analysis in ‘Shuchazao’ revealed tissue-biased expression and divergent drought responses, with A, S, I and M subfamilies showing stronger PEG-induced responsiveness. Together, these results establish a pan-genome-informed *bZIP* resource and highlight CNV, duplication mode and dispensable gene variation as potential drivers of tea *bZIP* diversification.

## Introduction

Tea (*Camellia sinensis*) is one of the most widely consumed non-alcoholic beverages worldwide and is valued for both its distinctive flavor and potential health-promoting properties, which are mainly attributed to bioactive compounds such as catechins, theanine, caffeine, and other polyphenols (Kumar et al., 2025; Payne et al., 2025). In addition to its health-promoting properties, tea is also an economically important crop and commercial beverage, with the global tea market projected to exceed USD 318 billion by 2025 (Radeva-Ilieva et al., 2025).

Plant transcription factors are central regulators that control spatiotemporal gene expression and coordinate plant growth, development and environmental adaptation (Kaur and Rishi, 2025). Among them, basic leucine zipper (*bZIP*) transcription factors constitute one of the most extensively studied TF families in plants and are characterized by a conserved *bZIP* domain, typically 60–80 amino acids in length, composed of an N-terminal basic region responsible for DNA binding and a C-terminal leucine zipper that mediates homo- or heterodimer formation; these structural features allow *bZIP* proteins to recognize ACGT-containing cis-elements, such as ABRE and G-box motifs, and to function as transcriptional activators or repressors in diverse signaling pathways (Xiao et al., 2021; Liu et al., 2023). Plant *bZIP* genes are generally divided into multiple phylogenetic subgroups, such as A–K, M and S in Arabidopsis, and different subgroups have undergone functional diversification during plant evolution (Feng et al., 2025). Subgroup A members, including ABI5/ABF/AREB-type genes, are closely associated with ABA-dependent signaling and play key roles in seed germination, stomatal regulation, drought tolerance and salt-stress responses; recent studies further showed that soybean Gm*bZIP*60 and Gm*bZIP*59 positively regulate salt and drought tolerance by activating stress- and hormone-responsive genes (Chai et al., 2025a, 2025b). Subgroup S1 *bZIP*s, often acting together with subgroup C members, serve as metabolic and developmental regulators by integrating sugar, amino-acid and energy signals, thereby affecting seed maturation, root growth, flowering, fruit quality and stress responses; for example, Pp*bZIP*44 was reported to regulate carbohydrate metabolism, amino-acid metabolism and flavonoid biosynthesis in pear and tomato fruits (Wang et al., 2021, 2023). Other *bZIP* subgroups also have specialized biological functions: HY5-type *bZIP*s participate in light-mediated photomorphogenesis, root development, pigment biosynthesis, hormone responses and temperature adaptation, whereas TGA-type *bZIP*s are involved in plant growth, defense and stress-related hormone signaling (Tao et al., 2024). In addition, ER stress-related *bZIP*s, such as *bZIP*17, *bZIP*28 and *bZIP*60, link unfolded protein response signaling with growth regulation and thermomorphogenesis, indicating that *bZIP* transcription factors act as important molecular hubs connecting developmental programs with abiotic stress adaptation (Lu et al., 2024; Zhang et al., 2025).

To date, *bZIP* transcription factors have been identified in several model plant species and important economic crops, including *Arabidopsis thaliana* (Jakoby et al., 2002), rice (Nijhawan et al., 2008), rapeseed (Zhou et al., 2017), tomato (Li et al., 2015), potato (Kumar et al., 2021), Chinese cabbage (Bai et al., 2016), and grape (Liu et al., 2014), providing important references for understanding the evolution and functional divergence of the *bZIP* gene family. Although tea plant genomic resources have expanded rapidly in recent years, with multiple chromosome-level assemblies and pangenome datasets becoming available, current studies on the *bZIP* transcription factor family in tea plant remain largely limited to genome-wide identification and expression analysis based on a single reference genome (Xia et al., 2020; Chen et al., 2023b; Kong et al., 2025). Therefore, a cross-genome comparative analysis of the tea plant *bZIP* family is still lacking, which limits our understanding of its evolutionary conservation, copy number variation, and potential functional diversification among different tea germplasms (Zhang et al., 2024). Here, by integrating 21 C*. sinensis* genomes, we performed a systematic comparative analysis of the *bZIP* transcription factor family to clarify its evolutionary dynamics and to provide a valuable genomic resource for future functional studies of organ development and trait improvement in *C. sinensis*.

## Materials and methods

### Data sources

Protein sequences, coding DNA sequences (CDS), and GFF3 annotation file for 21 C*. sinensis* were downloaded from the Tea-Pangenome database (https://www.tea-pangenome.cn/) (Chen et al., 2023b). Genome assembly completeness for the 21 C*. sinensis* genomes was further evaluated using BUSCO, and the resulting completeness metrics were summarized in **Supplementary Table 3** to provide a quality-control reference for the subsequent pangenomic analyses. The transcriptome expression matrices for SCZ (Shuchazao, a *C. sinensis* species) were downloaded from the Tea Plant Information Archive (TPIA; http://tpia.teaplants.cn/) (Xia et al., 2019). The *bZIP* domain HMM profiles (PF00170) were obtained from the InterPro database (http://www.ebi.ac.uk/) (Mulder and Apweiler, 2008). Protein sequences and Genome sequences for 1015 plant genome were downloaded from PLANTGIR database (Liu et al., 2024).

### *bZIP* family identification and physicochemical property analysis

To comprehensively identify *bZIP* gene family members in the target genome, a combined BLASTP- and hidden Markov model (HMM)-based strategy was performed (Sun et al., 2026). First, the whole-genome protein sequences of the target species were obtained from the genome annotation file, and only the longest protein isoform (non-redundant *bZIP* genes) was retained for each gene when alternative transcripts were present. Known *bZIP* protein sequences from *A. thaliana* were collected and used as queries to search against the target protein database using BLASTP with an E-value cutoff of 1e−5 (Camacho et al., 2023). In parallel, the HMM profiles of the *bZIP* domains (PF00170), were downloaded from the InterPro database and used to scan the target proteome with HMMER 3.0 (Sinha and Lynn, 2014). Candidate *bZIP* proteins obtained from the BLASTP and HMM searches were merged, and redundant sequences were removed. All candidate proteins were then submitted to InterProScan for domain confirmation. Proteins lacking a conserved *bZIP* domain or containing incomplete domain regions were excluded from subsequent analyses. Physicochemical properties of the *bZIP* genes were predicted using the Protein Parameter Calc module in TBtools-II (Chen et al., 2023a).

### Phylogenetic analysis of *bZIP* genes

The 21 *bZIP* protein sequences identified from *C. sinensis* were combined with previously reported *A. thaliana bZIP* protein sequences to determine their phylogenetic relationships and subgroup classification. All *bZIP* protein sequences were aligned using MAFFT v7.475 (Rozewicki et al., 2019). The resulting multiple sequence alignment was used to construct a maximum-likelihood phylogenetic tree with IQ-TREE v2.4.0 (Minh et al., 2020), in which the best-fitting amino acid substitution model was automatically selected, and branch support was evaluated using 1,000 bootstrap replicates. The *C. sinensis bZIP* proteins were assigned to corresponding *bZIP* subgroups according to their clustering relationships with the well-characterized *A. thaliana bZIP* members and previously reported *bZIP* classifications. The final phylogenetic tree was visualized and annotated using iTOL (https://itol.embl.de/) (Letunic and Bork, 2024).

### Pangenome analysis of the *bZIP* gene family

Orthogroup inference for the *bZIP* gene set from the 21 C*. sinensis* genomes was performed using OrthoFinder v2.5.4 (Emms and Kelly, 2019). The identified *bZIP* protein sequences from all *C. sinensis* genomes were used as input to infer orthologous groups. Based on their presence or absence across the 21 genomes, *bZIP* genes were classified as core genes if they were detected in all genomes, whereas genes absent from at least one genome were defined as dispensable genes. The dispensable orthogroups contains softcore orthogroups (with a lower cut-off of 90%, hence conserved in 19 or 20 of the 21 cultivars), shell orthogroups (with a lower cut-off of 10%, hence conserved in 3 to 18 of the 21 cultivars, and specific orthogroups (<10% hence in 1 or 2 of the 21 cultivars) (Tong et al., 2025). The resulting orthogroup information was further used to evaluate the conservation and variation of the *bZIP* gene family among *C. sinensis* genomes (Tong et al., 2025).

### Gene duplication-type analysis

To investigate the duplication patterns of the *bZIP* gene family, whole-genome protein sequences from the *C. sinensis* genomes were first subjected to all-versus-all sequence similarity searches using DIAMOND with an E-value cutoff of ≤ 1e−5 (Buchfink et al., 2015). The resulting similarity files, together with the corresponding genome annotation files converted into MCScanX-compatible format, were used as input for MCScanX. Gene duplication types were then classified using the duplicate_gene_classifier program implemented in MCScanX (Wang et al., 2024). Based on the genome-wide classification results, the identified *bZIP* genes were extracted and assigned to different duplication categories, including singleton, dispersed, proximal, tandem, and Whole Genome Duplication (WGD)/segmental duplicates.

### Selection pressure analysis

To evaluate the selection pressure acting on the *bZIP* gene family, coding sequences and corresponding protein sequences of *bZIP* genes from the 21 C*. sinensis* genomes were used as input files. Homologous *bZIP* gene pairs within each orthogroup were extracted based on the OrthoFinder results. The nonsynonymous substitution rate (Ka), synonymous substitution rate (Ks), and Ka/Ks ratio of each homologous gene pair were calculated using the Simple Ka/Ks Calculator module in TBtools-II (Chen et al., 2023a). Gene pairs that failed to generate valid Ka or Ks estimates were excluded from subsequent analyses.

### PCC-based co-expression network analysis

To investigate the coordinated expression patterns of SCZ *bZIP* genes under PEG-induced stress, Pearson correlation coefficient (PCC) analysis was performed based on their expression profiles using a custom Python script (Aoki et al., 2007; Zainal-Abidin et al., 2022). Prior to correlation analysis, the expression values of SCZ *bZIP* genes under PEG treatment were transformed using log2(x + 1). Pairwise PCC values were calculated for all possible *bZIP* gene pairs, and only strongly correlated pairs with |PCC| ≥ 0.95 were retained for co-expression network construction. Gene pairs with PCC > 0 and PCC < 0 were considered positively and negatively correlated, respectively. The resulting co-expression network was imported into Gephi for visualization and network topology analysis (Bastian et al., 2009).

### Data processing

All statistical tests were performed using Python scripts, and all figures were generated in R scripts.

## Results

### Identification of *bZIP* family in 1015 plant genomes

Based on 1,015 plant genomes, 81,340 *bZIP* genes were identified across eight major plant clades (**Supplementary Table 1**). The *bZIP* genes were widely present in the vast majority of species studied, with only one algal species lacking *bZIP* genes. Across all species, the median *bZIP* family size was 69 genes per genome, with a copy number range of 0–563, indicating that *bZIP* represents a widely conserved but unevenly expanded transcription factor family in plants.

**Figure 1:**
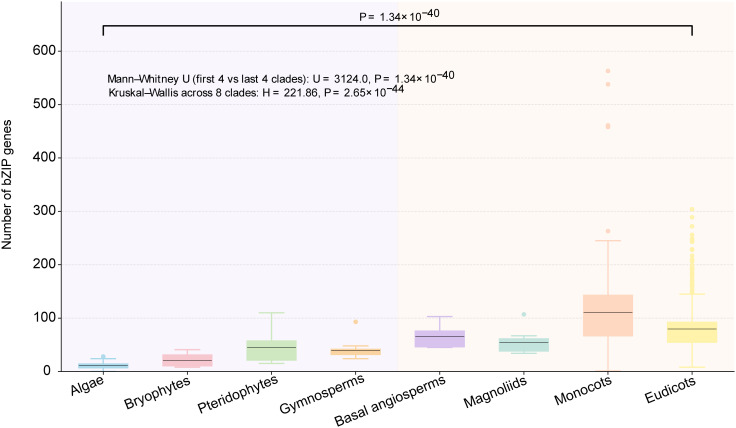
Distribution of *bZIP* gene copy numbers among eight major plant clades.

Clear differences in *bZIP* copy number were observed among major evolutionary lineages. Algae contained relatively small *bZIP* repertoires, with a median of 10 genes and an IQR of 7–14 (**Figure 1**, **Supplementary Table 2**). Bryophytes showed a modest increase, with 8–41 *bZIP* genes per species and a median of 15.5. In vascular plants, *bZIP* copy numbers increased further. Pteridophytes had a median of 38 genes, while gymnosperms showed a similar median of 36 genes, suggesting that the expansion of the *bZIP* family had already initiated before the diversification of seed plants. Angiosperms exhibited substantially larger *bZIP* repertoires than early-diverging plant lineages. Early diverging angiosperms and magnoliids had median values of 48 and 51 genes, respectively, showing higher *bZIP* copy numbers than non-angiosperm lineages but lower copy numbers than monocots and eudicots. Among the two major angiosperm groups, monocots displayed the highest *bZIP* copy numbers, with a median of 84 genes, a mean of 110.37 genes, and a maximum of 563 genes. Eudicots also showed extensive *bZIP* expansion, with a median of 69 genes, a mean of 79.64 genes, and a maximum of 304 genes (**Figure 1**; **Supplementary Table 2**).

The median bZIP copy numbers increased from 10 in algae to 15.5 in bryophytes, 36 in pteridophytes plus gymnosperms, and 71 in angiosperms. A Kruskal–Wallis test showed significant differences among these four groups (H = 221.86, p = 2.65 × 10^−44^). To further assess whether bZIP gene numbers were significantly elevated in angiosperms, the sampled species were subsequently divided into two broad evolutionary groups: non-angiosperms, comprising algae, bryophytes, pteridophytes, and gymnosperms, and angiosperms, comprising early diverging angiosperms, magnoliids, monocots, and eudicots. Pairwise Mann–Whitney U tests with Benjamini–Hochberg correction further showed that angiosperms contained significantly more bZIP genes than algae, bryophytes, and pteridophytes plus gymnosperms. In addition, Spearman correlation analysis detected a significant positive association between the ordered major plant clades used in this study and *bZIP* copy number (ρ = 0.421, p = 7.12 × 10^−45^) (**Figure 1**; **Supplementary Table 2**). These statistical results support the conclusion that the *bZIP* gene family underwent significant expansion during plant evolution, with the strongest expansion occurring in angiosperms.

### Identification and phylogenetic analysis of the *bZIP* gene family in 21 *Camellia sinensis*

A genome-wide survey was performed to identify *bZIP* transcription factor genes in 21 tea genomes (**Supplementary Table 3**). In total, 1,635 non-redundant *bZIP* genes were identified, with the number of *bZIP* members varying from 73 to 88 among different genomes (**Supplementary Table 4**). The average number of *bZIP* genes was 77.9 per genome, with a median of 77, indicating that the *bZIP* family size is generally conserved across the sampled tea genomes. Among them, WYSX contained the largest number of *bZIP* genes (88), followed by MSBH (87) and JGY (83), whereas GH3H, JMZ, and JX had the smallest *bZIP* repertoires, each containing 73 members. Most tea genomes harbored 74–81 *bZIP* genes, suggesting limited copy number variation within this gene family.

**Figure 2:**
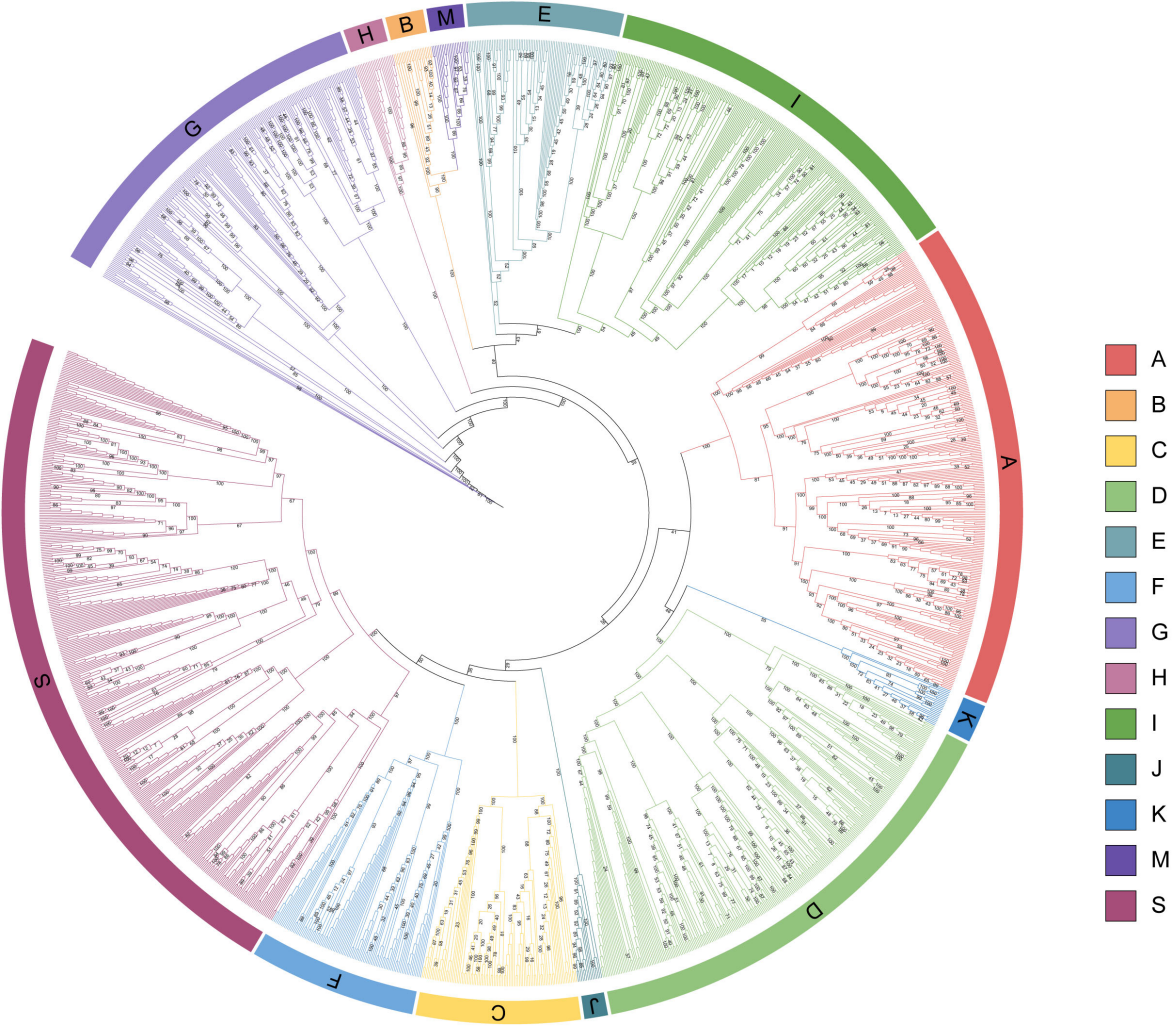
Phylogenetic analysis. A phylogenetic tree constructed from *bZIP* family genes of 21 C*. sinensis* and *A. thaliana*. Bootstrap values were set to 1000 and are indicated on the branches of the phylogenetic tree.

To further clarify the evolutionary relationships of the identified *bZIP* genes, the 1,635 *bZIP* members from 21 tea genomes were classified into 13 subfamilies, including A, B, C, D, E, F, G, H, I, J, K, M, and S (**Figure 2**). Among all subfamilies, subfamily S contained the largest number of members, with 371 genes, accounting for 22.7% of all identified *bZIP* genes. This was followed by subfamilies A, D, I, and G, which contained 259, 245, 202, and 193 genes, respectively (**Supplementary Table 5**). These five subfamilies together represented the major components of the tea *bZIP* family. In contrast, several subfamilies contained relatively few members, including H, M, and K, each with 22 genes, B with 20 genes, and J with only 14 genes, indicating that these subfamilies were more conserved or experienced limited expansion. At the species level, most major subfamilies were consistently retained across all 21 tea genomes, especially subfamilies S, A, D, I, G, F, C, and E, suggesting a high degree of evolutionary conservation. However, minor subfamilies showed partial absence in some genomes; for example, subfamilies B and H were absent in two genomes, whereas subfamily J was absent in seven genomes. The copy number of subfamily S ranged from 14 to 21 among different tea genomes, with the highest values observed in SCZ and WYSX, while subfamily A ranged from 9 to 15 members, with JGY and MSBH containing the largest number of A-subfamily genes (**Supplementary Table 6**). Overall, the phylogenetic classification revealed that the tea *bZIP* family is composed of several broadly conserved major subfamilies and a small number of low-copy subfamilies, reflecting both evolutionary conservation and subfamily-specific diversification.

### Physicochemical property analysis of the *bZIP* gene family

The physicochemical properties of the 1,635 identified tea *bZIP* proteins were further analyzed to characterize their basic protein features. The predicted molecular weights varied widely from 7.92 to 222.56 kDa, with an average of 36.46 kDa and a median of 36.70 kDa (**Supplementary Table 7**). The theoretical isoelectric points ranged from 4.85 to 11.56, with a mean value of 7.24 and a median value of 6.71 (**Supplementary Table 7**). Among these proteins, 909 members showed acidic properties with pI values below 7.0, whereas 708 members were basic proteins, suggesting that the tea *bZIP* family contains both acidic and basic members but is slightly biased toward acidic proteins. The instability index ranged from 31.12 to 84.31, with an average of 56.13, and 1,539 proteins showed instability index values greater than 40, implying that most tea *bZIP* proteins are predicted to be unstable (**Supplementary Table 7**). The aliphatic index ranged from 38.54 to 98.37, with an average of 68.94 (**Supplementary Table 7**). In addition, the GRAVY values ranged from −1.37 to 0.11, with a mean of −0.70, and 1,628 proteins had negative GRAVY values, indicating that nearly all tea *bZIP* proteins are hydrophilic (**Supplementary Table 7**).

**Figure 3:**
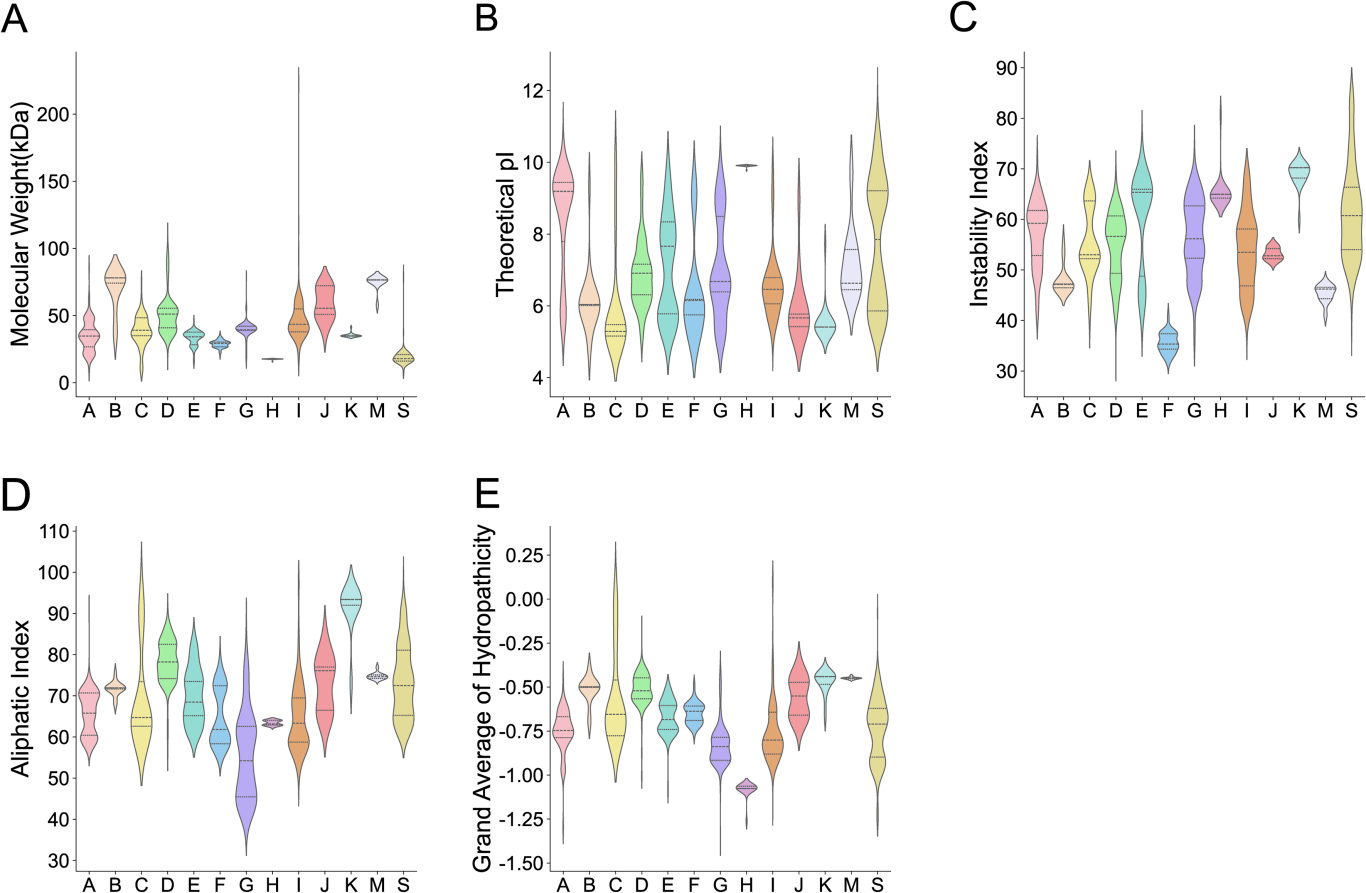
Physicochemical property analysis. **(A)** Distribution of molecular weight among *bZIP* subfamilies. **(B)** Distribution of theoretical isoelectric point (pI) among *bZIP* subfamilies. **(C)** Distribution of instability index among *bZIP* subfamilies. **(D)** Distribution of aliphatic index among *bZIP* subfamilies. **(E)** Distribution of grand average of hydropathicity (GRAVY) among *bZIP* subfamilies.

Subfamily-level physicochemical analysis revealed clear differences among the tea *bZIP* groups (**Figure 3**). The mean molecular weight varied markedly among subfamilies, ranging from 17.55 kDa in subfamily H and 19.19 kDa in subfamily S to 69.90 kDa in subfamily B and 74.82 kDa in subfamily M (**Figure 3A**; **Supplementary Table 8**). The theoretical pI also differed among subfamilies. Subfamilies K, C, J and B showed lower mean pI values of 5.53, 5.89, 5.94 and 6.11, respectively, suggesting predominantly acidic proteins, while subfamilies A and H showed higher mean pI values of 8.45 and 9.90, respectively, indicating more basic protein characteristics (**Figure 3B**; **Supplementary Table 8**). The instability index ranged from 35.55 in subfamily F to 68.99 in subfamily K. Subfamily F was the only group with an average instability index below 40, suggesting relatively stable protein properties, whereas most other subfamilies, especially K, H, S and E, showed higher instability index values (**Figure 3C**; **Supplementary Table 8**). The aliphatic index was highest in subfamily K, with a mean value of 91.37, followed by D, M, S and J, while subfamily G showed the lowest mean aliphatic index of 55.66 (**Figure 3D**; **Supplementary Table 8**). All subfamilies had negative mean GRAVY values, ranging from −1.08 in subfamily H to −0.45 in subfamily M, with subfamily H being the most hydrophilic and subfamily M the least hydrophilic (**Figure 3E**; **Supplementary Table 8**). The coefficient of variation further showed that different physicochemical properties varied unevenly among subfamilies. Molecular weight exhibited the highest variation in subfamilies S, I and A, with CV values of 40.66%, 37.34% and 32.71%, respectively, whereas subfamilies H, K and M showed much lower variation, indicating more conserved protein sizes. For theoretical pI, subfamilies C, S and F showed relatively high CV values, while subfamily H had an extremely low CV of 0.28%, suggesting conserved basic properties within this group (**Supplementary Table 8**). The instability index was most variable in subfamilies E, S and I, but relatively conserved in J, K and M. The aliphatic index showed the greatest variation in subfamilies G and C, whereas H, M and B were more conserved. For GRAVY, absolute CV values indicated that hydropathicity varied most strongly in subfamily C, followed by S, I and J, while subfamilies M and H showed the lowest variation. Overall, these results suggest that tea *bZIP* subfamilies differ substantially in protein size, charge, stability, thermostability-related composition and hydrophilicity, and the CV patterns indicate that some subfamilies, such as H, K and M, are relatively physicochemical property conserved, whereas S, I, C and A show stronger internal diversification.

### Core and dispensable *bZIP* gene analysis in 21 *Camellia sinensis* genomes

**Figure 4:**
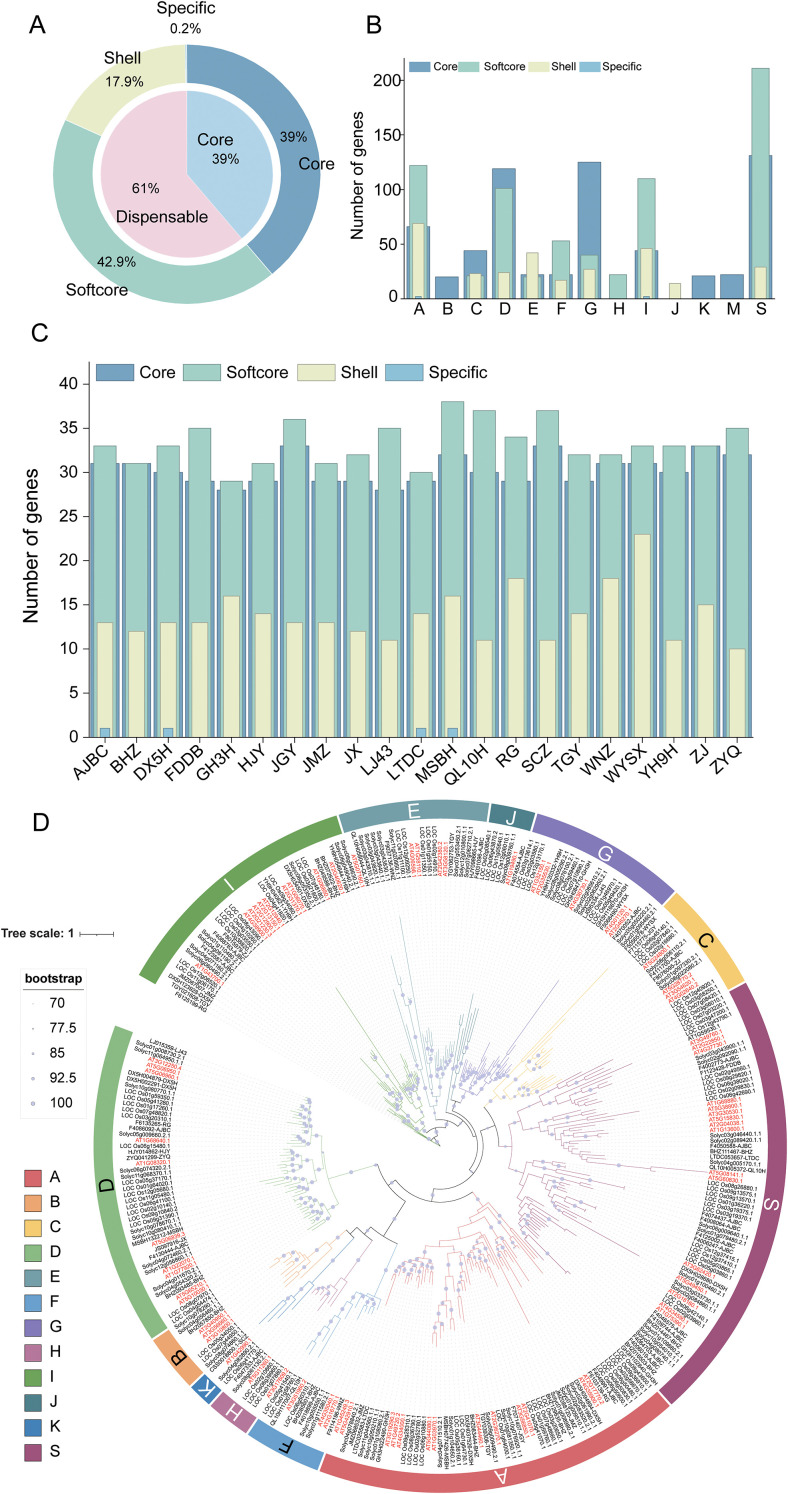
Core and dispensable (softcore, shell and specific) gene analysis. **(A)** Proportion of core and dispensable (softcore, shell and specific) genes. **(B)** Numbers of core and dispensable (softcore, shell and specific) genes in each subfamily. **(C)** Column chart of core and dispensable (softcore, shell and specific) genes across 21 C*. sinensis*. **(D)** OGG-level phylogenetic tree constructed using one representative tea *bZIP* protein from each of the 77 orthogroups together with *bZIP* proteins from *A. thaliana*, *O. sativa*, and *S. lycopersicum*.

To investigate the composition of core and dispensable *bZIP* genes in tea plants from an orthologous perspective, OrthoFinder was used to cluster the 1,635 *bZIP* genes into orthogroups. A total of 1,631 *bZIP* genes were assigned to 77 orthogroups (OGGs), including 22 core orthogroups comprising 636 genes, which accounted for 39% of the clustered *bZIP* genes (**Supplementary Table 9**). In addition, 55 dispensable orthogroups were identified, containing 995 genes and representing 61% of the clustered *bZIP* genes (**Figure 4A**; **Supplementary Table 10**). The 55 dispensable orthogroups were further classified into 32 softcore orthogroups containing 700 genes, 21 shell orthogroups containing 291 genes, and 2 specific orthogroups containing 4 genes. Notably, the four specific genes were each detected in a single tea cultivar, suggesting their potential contribution to cultivar-specific traits (**Figure 4C**). It should be noted that softcore orthogroups conceptually occupy an intermediate position between strictly core and clearly dispensable orthogroups. Because the BUSCO completeness of the analyzed tea genomes ranged from 87.1% to 93.9% (**Supplementary Table 3**), the absence of orthologues from one or two genomes may reflect either true biological presence–absence variation or residual genome/annotation incompleteness.

To further place these pangenome-defined orthogroups within a broader evolutionary context, we constructed an OGG-level phylogenetic tree using one representative tea *bZIP* protein from each of the 77 OGGs, together with *bZIP* proteins from *A. thaliana*, *Oryza sativa*, and *Solanum lycopersicum* (**Figure 4D**). The representative tea sequences were assigned to 12 bZIP subfamilies, indicating that the tea *bZIP* pangenome covers most of the major *bZIP* lineages detected in this analysis. After annotating the tea sequences according to their pangenome categories, we found that core OGGs were preferentially associated with conserved cross-species *bZIP* lineages (**Figure 4B**). For example, tea OGGs belonging to the B and K subfamilies were consistently classified as core components, while the G subfamily also showed a high proportion of core genes, suggesting that these lineages are broadly retained across tea genomes and conserved relative to the reference species. In contrast, softcore, shell, and specific OGGs were unevenly distributed among subfamilies and were more frequently observed in several expanded or variable lineages, such as A, I, E, S, and J. Notably, shell genes were enriched in A, I, and E, whereas the J subfamily was entirely composed of shell genes, and specific OGGs were restricted to the A and I subfamilies. These results indicate that dispensable variation in the tea *bZIP* family is not randomly distributed across the phylogeny but is concentrated in particular subfamilies.

Consistent with the OGG-level phylogenetic framework, the proportions of core and dispensable *bZIP* genes differed markedly among subfamilies (**Figure 4B**). Subfamilies B, K, and M were entirely composed of core genes, with all members assigned to core orthogroups. Subfamily G also showed a high core-gene proportion, with 125 of 192 genes classified as core genes, accounting for 65.1% of this subfamily (**Supplementary Table 12**). In contrast, several large subfamilies were dominated by dispensable genes. For example, subfamily I contained 158 dispensable genes out of 202 members, representing 78.2% of the subfamily, followed by subfamilies F, A, E, and S, in which dispensable genes accounted for 76.1%, 74.5%, 73.8%, and 64.7%, respectively. Subfamilies C and D displayed relatively balanced core and dispensable compositions, with core genes accounting for 50.0% and 48.8% of their members, respectively. Further subdivision of the dispensable component revealed that softcore genes represented the major variable fraction in most expanded subfamilies, especially S, A, I, and D, which contained 211, 122, 110, and 101 softcore genes, respectively. Shell genes were unevenly distributed among nine subfamilies, including A, I, E, S, G, D, C, F, and J. Among the 291 shell genes, subfamily A contained the largest number, with 69 shell genes, accounting for 23.7% of all shell genes and 26.6% of the A subfamily. This was followed by subfamily I, with 46 shell genes, accounting for 15.8% of all shell genes and 22.8% of the I subfamily, and subfamily E, with 42 shell genes, accounting for 14.4% of all shell genes and 50.0% of the E subfamily. Although subfamily J contained only 14 shell genes, all of its members were classified as shell genes, representing 100% of this subfamily. In contrast, subfamily H contained 22 genes, all of which were assigned to the softcore category. The four specific genes were restricted to subfamilies A and I, with two genes in each subfamily, accounting for 0.8% and 1.0% of the A and I subfamilies, respectively (**Supplementary Table 12**). These results indicate that the tea *bZIP* family contains both conserved core subfamilies and dynamically variable dispensable subfamilies, with shell genes mainly enriched in A, I, and E, and cultivar-specific genes confined to A and I, suggesting that these expanded and variable subfamilies may contribute more strongly to cultivar-level functional divergence in tea plants.

### Duplication type and CNV analysis of the *bZIP* gene family in 21 *Camellia sinensis* genomes

To explore the evolutionary mechanisms underlying the expansion of the *bZIP* gene family in *C. sinensis*, we classified all identified *bZIP* genes into five duplication categories, including singleton, dispersed, proximal, tandem, and WGD/segmental duplicates. Among the 1,635 classified *bZIP* genes, WGD/segmental duplicates represented the largest proportion, accounting for 968 genes (59.20%), followed by dispersed duplicates with 526 genes (32.17%) (**Supplementary Table 13**). In contrast, proximal, tandem, and singleton genes contributed only minor fractions, with 57 (3.49%), 46 (2.81%), and 38 (2.32%) genes, respectively. These results indicate that large-scale duplication events, particularly WGD/segmental duplication, were the predominant force driving the expansion and retention of the *bZIP* gene family in *C. sinensis*.

**Figure 5:**
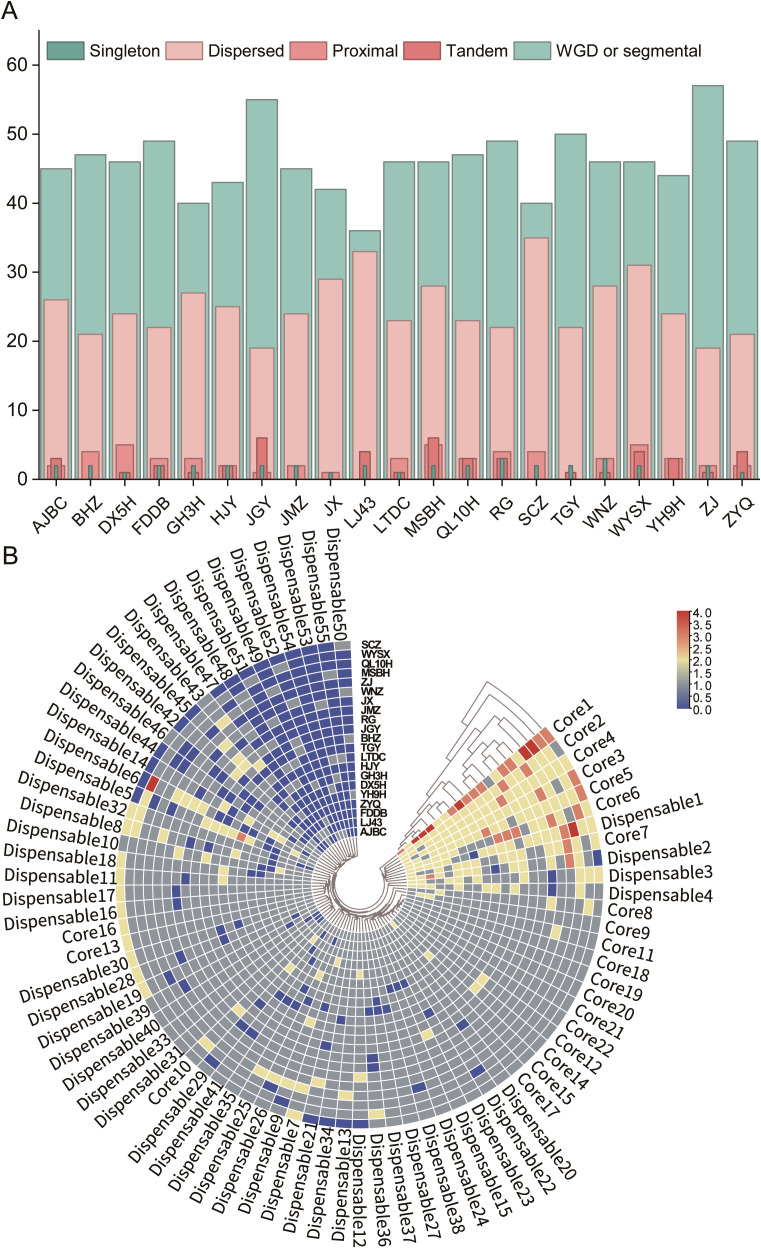
Gene duplication mode and CNV analysis. **(A)** Column chart showing the distribution of gene duplication modes across 21 C*. sinensis*. The y-axis indicates the number of genes. **(B)** Heatmap of copy number variation (CNV); orthogroups are shown on the outer periphery, and species abbreviations are displayed on the inner radial axes.

At the species level, WGD/segmental duplication was the dominant duplication mode in all 21 C*. sinensis* genomes, ranging from 48.00% in LJ43 to 70.37% in ZJ, suggesting that the contribution of WGD/segmental duplication to *bZIP* family expansion was broadly conserved across *C. sinensis* (**Figure 5A**; **Supplementary Table 13**). Dispersed duplicates represented the second major category in most species, with proportions ranging from 22.89% in JGY to 44.00% in LJ43, indicating that dispersed duplication also contributed to lineage-specific retention of *bZIP* genes. By contrast, singleton, proximal, and tandem duplicates were present at relatively low frequencies in most genomes. At the subfamily level, distinct duplication patterns were observed among *bZIP* subfamilies. WGD/segmental duplicates were highly represented in subfamilies G, S, E, I, and D, accounting for 77.72%, 76.01%, 70.24%, 64.85%, and 64.08% of the corresponding subfamilies, respectively, indicating that these subfamilies were mainly shaped by large-scale duplication. In contrast, subfamilies J, B, C, and M were dominated by dispersed duplicates, with proportions of 92.86%, 80.00%, 78.65%, and 68.18%, respectively (**Supplementary Table 13**). Notably, subfamilies K and H were mainly composed of singleton genes, accounting for 95.45% and 68.18%, respectively, suggesting limited duplication-mediated expansion in these groups. Subfamily F showed a relatively high contribution of local duplication, including proximal and tandem duplicates, which accounted for 18.48% and 19.57%, respectively, implying that small-scale duplication may have played a more important role in the expansion of this subfamily.

We further compared duplication-type composition between core and dispensable *bZIP* genes to determine whether specific duplication modes were preferentially associated with different pangenome gene categories. Among the 1,631 *bZIP* genes assigned to core or dispensable categories, core genes contained 21 singleton, 257 dispersed, 17 proximal, 8 tandem, and 333 WGD/segmental genes, whereas dispensable genes contained 16 singleton, 267 dispersed, 39 proximal, 38 tandem, and 635 WGD/segmental genes (**Supplementary Table 15**). A chi-square test revealed a significant difference in duplication-type composition between core and dispensable genes (χ^2^ = 46.53, df = 4, *P* = 1.91 × 10^−9^). Pairwise Fisher’s exact tests with Benjamini–Hochberg correction further showed that singleton and dispersed genes were significantly enriched in the core set (singleton: odds ratio = 2.09, adjusted *P* = 0.034; dispersed: odds ratio = 1.85, adjusted *P* = 7.22 × 10^−8^), whereas tandem and WGD/segmental duplicates were significantly enriched in the dispensable set (tandem: odds ratio = 0.32, adjusted *P* = 0.0032; WGD/segmental: odds ratio = 0.62, adjusted *P* = 1.32 × 10^−5^). Proximal duplicates showed no significant enrichment in either category (adjusted *P* = 0.210). These results suggest that dispersed duplication contributed more strongly to the maintenance of conserved core *bZIP* genes, whereas tandem and WGD/segmental duplication preferentially promoted the expansion of dispensable *bZIP* genes, potentially facilitating lineage-specific diversification in *C. sinensis*.

Copy-number variation (CNV) was further classified to distinguish different patterns of *bZIP* orthogroup variation among the 21 C*. sinensis* genomes. Among the 77 *bZIP* OGGs, only five OGGs (6.49%; OG0000030, OG0000032, OG0000037, OG0000038, and OG0000039) were strictly retained as single-copy groups across all genomes, whereas the remaining 72 OGGs (93.51%) showed CNV (**Figure 5B**; **Supplementary Table 10**). These CNV OGGs included 17 expansion-only OGGs, 25 absence-only OGGs, and 30 mixed CNV OGGs, indicating that both copy-number expansion and gene absence contributed to *bZIP* orthogroup variation. The degree of copy-number expansion was generally moderate. Among the 72 CNV OGGs, 36 OGGs expanded to a maximum of two copies, eight OGGs expanded to three copies, and three OGGs expanded to four copies in at least one genome, while no OGG exceeded four copies. All 17 expansion-only OGGs belonged to the core category, indicating that some universally present *bZIP* orthogroups varied in copy dosage while remaining retained across all 21 genomes. Among them, OG0000000 showed the strongest expansion, with 62 total copies and up to four copies in FDDB, LTDC, MSBH, QL10H, YH9H, and ZYQ. Several other core OGGs, including OG0000001–OG0000005 and OG0000007, also showed moderate expansion, reaching up to three copies in specific genomes. In contrast, non-core OGGs were mainly characterized by presence–absence variation, with or without additional copy-number expansion. All 55 non-core OGGs showed CNV, including 30 mixed CNV OGGs and 25 absence-only OGGs. Softcore OGGs mostly showed mild presence–absence variation combined with occasional expansion. For example, OG0000006 was present in 20 genomes, absent from HJY, and expanded to four copies in QL10H, whereas OG0000008 was present in 19 genomes, absent from SCZ and TGY, and expanded to three copies in FDDB and MSBH. Shell and specific OGGs showed stronger absence patterns. For instance, shell OG0000014 was retained in only 15 genomes, absent from six genomes, and expanded to four copies in WYSX. The two specific OGGs, OG0000075 and OG0000076, were each retained in only two genomes and occurred as single-copy orthogroups, suggesting rare or highly lineage-restricted retention.

At the genome level, CNV patterns also differed among accessions. SCZ contained the largest number of expanded OGGs (20) and also the highest number of absent OGGs (19), suggesting relatively strong *bZIP* orthogroup turnover in this accession. In contrast, WYSX and MSBH retained high total *bZIP* copy numbers, each with 87 genes assigned to OGGs, but showed fewer absent OGGs, with eight and ten absent OGGs, respectively, suggesting expansion-biased retention rather than broad gene loss. Overall, these results indicate that CNV is widespread in the *C. sinensis bZIP* family, with core OGGs mainly showing copy-number dosage variation and non-core OGGs showing stronger presence–absence variation. These CNV patterns provide candidate orthogroups for future studies of cultivar-specific *bZIP* diversification, although direct functional or trait associations require further experimental validation.

### Selection pressure analysis of the *bZIP* gene family in 21 *Camellia sinensis* genomes

**Figure 6:**
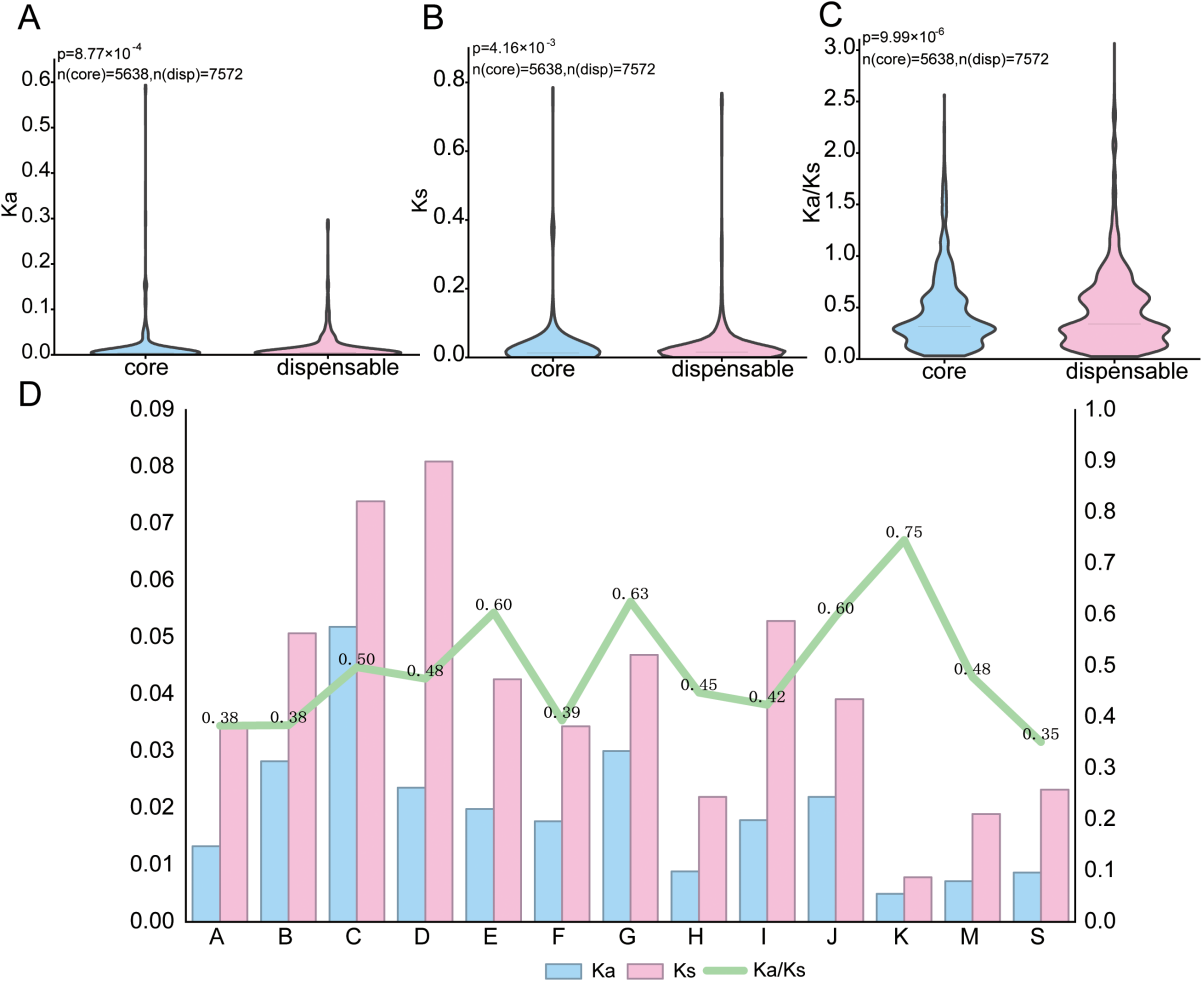
Selection pressure analysis. **(A)** Violin plot of Ka values for core and diapensable genes (Mann-Whitney U test). **(B)** Violin plot of Ks values for core and diapensable genes (Mann-Whitney U test). **(C)** Violin plot of Ka/Ks values for core and dispensable genes (Mann-Whitney U test). **(D)** Mean Ka, Ks, and Ka/Ks values across *bZIP* subfamilies.

To evaluate the evolutionary constraints acting on the *bZIP* gene family across 21 C*. sinensis* genomes, we estimated the nonsynonymous substitution rate (Ka), synonymous substitution rate (Ks), and Ka/Ks ratio for 22,849 homologous gene pairs derived from OGGs (**Figure 6**; **Supplementary Table 16**). Among these pairs, 13,210 yielded statistically meaningful estimates. The great majority of homologous pairs showed evidence of purifying selection, with 12,226 pairs (92.55%) having Ka/Ks values below 1, whereas only 982 pairs (7.43%) exhibited Ka/Ks values greater than 1, suggesting possible positive selection (**Supplementary Table 16**). In addition, two homologous pairs had Ka/Ks values equal to 1, indicating that they may have evolved under neutral selection. To further compare selective constraints among different pangenome gene categories, homologous pairs were classified according to their genome-wide gene types, and only pairs with the same gene type at both ends were retained for subsequent analysis. The evolutionary rates of core and dispensable *bZIP* gene pairs were further compared using the Mann–Whitney U test. A total of 5,638 core gene pairs and 7,572 dispensable gene pairs with valid Ka, Ks, and Ka/Ks values were retained for analysis. The median Ka, Ks, and Ka/Ks values of core gene pairs were 0.0043, 0.0136, and 0.3172, respectively, whereas the corresponding values for dispensable gene pairs were slightly higher, at 0.0048, 0.0160, and 0.3398, respectively (**Figures 6A–C**). Mann–Whitney U tests revealed significant differences between core and dispensable gene pairs for Ka (p = 3.087 × 10^−11^), Ks (p = 5.7707 × 10^−22^), and Ka/Ks (p = 2.6497 × 10^−5^). However, the corresponding Cliff’s δ values were −0.067, −0.098, and −0.043, respectively, indicating negligible effect sizes. These results suggest that although dispensable *bZIP* gene pairs showed statistically higher substitution levels than core gene pairs, the magnitude of this difference was relatively small. Overall, these results indicate that *C. sinensis bZIP* genes are generally conserved under purifying selection, while some dispensable genes may have experienced modestly accelerated sequence evolution.

We further compared the mean Ka, Ks, and Ka/Ks values among the *bZIP* subfamilies to assess whether different subfamilies experienced distinct selective constraints (**Figure 6D**; **Supplementary Table 17**). The mean Ka/Ks values of all 13 subfamilies were lower than 1, ranging from 0.3514 to 0.7458, indicating that the *bZIP* gene family in *C. sinensis* was generally maintained under purifying selection. Nevertheless, the intensity of selective pressure varied among subfamilies. Subfamily K displayed the highest mean Ka/Ks value (0.7458), despite having relatively low mean Ka and Ks values of 0.0049 and 0.0078, respectively, suggesting a comparatively relaxed selective constraint within this group (**Supplementary Table 17**). Relatively high Ka/Ks values were also observed in subfamilies G, E, and J, with mean ratios of 0.6255, 0.6042, and 0.5971, respectively, implying a greater potential for sequence divergence and functional differentiation. In contrast, subfamilies S, A, B, and F showed lower mean Ka/Ks values of 0.3514, 0.3833, 0.3840, and 0.3938, respectively, suggesting stronger evolutionary conservation. Notably, subfamily C had the highest mean Ka value (0.0519), whereas subfamily D showed the highest mean Ks value (0.0809), indicating that these two subfamilies accumulated relatively more substitutions overall (**Supplementary Table 17**); however, their moderate Ka/Ks values still supported the predominance of purifying selection. Taken together, these results suggest that although purifying selection broadly shaped the evolution of *C. sinensis bZIP* genes, different subfamilies experienced unequal evolutionary constraints, which may have contributed to their functional diversification.

### Transcriptome analysis of the *bZIP* gene family in 21 *Camellia sinensis* genomes

**Figure 7:**
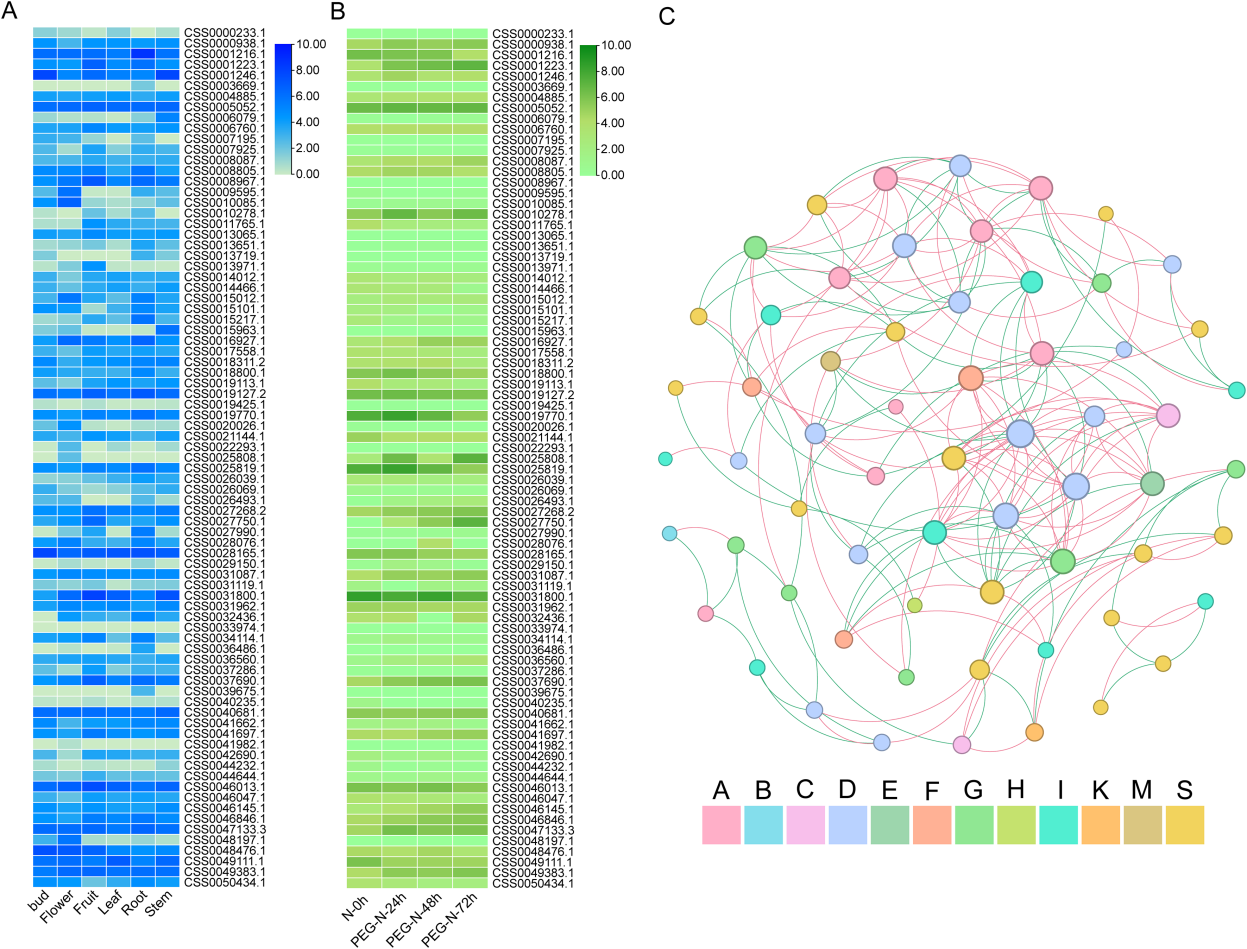
Transcriptome expression analysis across Shuchazao(SCZ). **(A)** Heatmap of transcriptome expression levels of 81 *bZIP* genes in six tissues of SCZ. **(B)** Heatmap of time-dynamic transcriptomic expression levels of 81 *bZIP* genes in SCZ under PEG-induced drought stress. **(C)** Co-expression network of SCZ *bZIP* genes under PEG-induced drought stress. Each node represents an individual SCZ *bZIP* gene, and node colors indicate the corresponding *bZIP* subfamilies. Edges connect pairs of *bZIP* genes showing strongly correlated expression profiles across the drought-treatment time points. Only gene pairs with |PCC| ≥ 0.95 were retained in the network. Red edges indicate positive correlations, whereas green edges indicate negative correlations.

The *C. sinensis* Shuchazao (SCZ) was selected as a representative species for transcriptome analysis. After log_2_(x+1) transformation of the expression values, the 81 *bZIP* genes showed broad but tissue-dependent expression across six tissues, with mean expression levels ranging from 2.983 in leaves to 3.773 in roots, followed by fruits (3.522), stems (3.463), flowers (3.273), and buds (3.176) (**Figure 7A**; **Supplementary Table 18**). At the subfamily level, several *bZIP* subfamilies displayed clear tissue-biased expression patterns. Subfamilies A, B, and G were preferentially expressed in fruits, with mean expression values of 4.076, 4.993, and 4.663, respectively, suggesting potential roles in fruit development. Subfamily C showed the highest expression in roots (5.610) and flowers (5.314), whereas subfamily D was mainly expressed in roots (3.588), indicating possible root-associated functions. Subfamily E exhibited a relatively restricted expression pattern, with higher expression in flowers (3.389) but much lower expression in fruits (0.273) and leaves (0.182). In contrast, subfamily F was broadly expressed across multiple tissues, particularly in stems (5.267), leaves (5.224), and fruits (5.056), suggesting a more constitutive expression pattern (**Supplementary Table 18**). Subfamilies I and S also showed root-preferential expression, with mean values of 4.491 and 3.612, respectively. We further compared the expression profiles of core and dispensable *bZIP* genes across tissues. Among the 81 genes. Core genes showed slightly higher mean expression than dispensable genes in all six tissues, including bud (3.403 vs. 3.020), flower (3.352 vs. 3.219), fruit (4.007 vs. 3.189), leaf (3.459 vs. 2.656), root (3.992 vs. 3.623), and stem (3.700 vs. 3.299) (**Supplementary Table 18**). This result suggests that core members may play more stable and broadly conserved roles in tissue development.

The 81 *bZIP* genes showed clear subfamily-dependent responses to PEG-induced drought stress (**Figure 7B**; **Supplementary Table 19**). Among the 12 detected subfamilies, subfamily A showed a gradual induction pattern, with mean expression increasing from 2.409 at 0 h to 3.029 at 24 h, 3.359 at 48 h, and 3.578 at 72 h, suggesting sustained drought responsiveness. Subfamily S also showed an induced expression pattern, increasing from 2.486 at 0 h to 2.979 at 24 h and remaining higher than the control at 48 h and 72 h. Subfamily I was moderately induced at early time points, with mean expression rising from 2.501 at 0 h to 2.944 at 24 h and 2.921 at 48 h, but returning to 2.479 at 72 h. In contrast, subfamilies C, D, and F showed decreased expression after drought treatment: subfamily C declined from 4.087 at 0 h to 3.824–3.916 under PEG treatment, subfamily D decreased from 2.265 to 1.777 by 72 h, and subfamily F decreased from 5.247 to 4.460 by 72 h, although F remained one of the most highly expressed subfamilies overall. Subfamily G displayed a transient response, peaking at 48 h (4.021), whereas subfamily E showed extremely low expression throughout the treatment, with values close to 0.000–0.184. The single-gene subfamilies K and M showed relatively high expression levels, with K peaking at 24 h (6.311) and M reaching the highest level at 72 h (5.473) (**Supplementary Table 19**). Overall, subfamilies A, S, I, and M appeared to be more responsive based on descriptive expression patterns, whereas subfamilies C, D, and F showed decreased expression levels after PEG treatment, suggesting that *bZIP* subfamilies exhibited different descriptive expression patterns under PEG-induced stress.

To identify coordinated PP2C expression patterns and prioritize candidate hub genes associated with the drought response, pairwise Pearson correlation coefficients (PCCs) were calculated using transcriptomic expression profiles under drought stress, and a stringent co-expression network was constructed by retaining gene pairs with |PCC| > 0.95 (**Figure 7C**; **Supplementary Table 20**). The resulting network comprised 62 PP2C genes and 200 edges, including 113 positive and 87 negative correlations, with all genes forming a single connected component. Inter-subfamily relationships predominated, accounting for 173 of the 200 edges, indicating extensive coordination among different PP2C subfamilies during drought stress (**Figure 7C**; **Supplementary Table 20**). Subfamily D formed the major network backbone, with its 14 genes participating in 99 edges, including 14 intra-subfamily connections. Subfamilies S and A also contributed substantially, with 15 and eight genes involved in 68 and 56 edges, respectively. The most frequent inter-subfamily connections were observed between subfamilies A and D (19 edges), D and S (18 edges), D and G (16 edges), and D and I (15 edges), further demonstrating the central connectivity of subfamily D (**Figure 7C**; **Supplementary Table 20**). Notably, the direction of intra-subfamily correlations differed among lineages: all six connections among subfamily A genes were positive, whereas six of the seven connections among subfamily S genes were negative, suggesting distinct patterns of coordinated expression within these subfamilies. Degree-based analysis identified three subfamily D genes as the most highly connected candidate hubs. CSS0015217.1 displayed the highest degree, with 15 edges comprising ten positive and five negative correlations, followed by CSS0011765.1 with 14 edges and CSS0044232.1 with 13 edges (**Figure 7C**; **Supplementary Table 20**). All three genes were connected with members from multiple subfamilies, highlighting their potential importance in the coordinated PP2C response to drought stress. In addition, CSS0021144.1 from subfamily G and CSS0019113.1 from subfamily F each formed 12 connections, indicating that highly connected candidate genes were also present outside subfamily D.

## Discussion

In this study, we performed a systematic evolutionary and pan-genomic analysis of the *bZIP* transcription factor family in plants, with a particular focus on 21 C*. sinensis* genomes. At the broad evolutionary scale, 81,340 *bZIP* genes were identified from 1,015 plant genomes, showing that *bZIP* genes are widely conserved across major plant lineages but have undergone marked copy-number expansion during plant evolution, especially in angiosperms (**Supplementary Table 1**). In tea plants, a total of 1,635 non-redundant *bZIP* genes were identified from 21 genomes, with 73–88 members per genome, suggesting that the overall size of the tea *bZIP* family is relatively conserved among different tea germplasms (**Supplementary Table 4**). Phylogenetic analysis classified these genes into 13 subfamilies, among which S, A, D, I and G represented the major expanded groups, whereas B, H, J, K and M were maintained as low-copy subfamilies (**Supplementary Table 5**). Pan-genomic orthogroup analysis further revealed that the tea *bZIP* family consisted of both conserved and variable components, including 22 core orthogroups and 55 dispensable orthogroups (**Supplementary Table 9**). Duplication-type analysis showed that WGD/segmental duplication was the dominant force driving *bZIP* expansion in tea plants, whereas CNV analysis indicated extensive copy-number variation across orthogroups (**Figure 5A**; **Supplementary Table 13**). Selection pressure analysis suggested that most homologous *bZIP* gene pairs evolved under purifying selection, while dispensable genes showed relatively relaxed constraints (**Supplementary Table 16**). Transcriptome analysis in Shuchazao further revealed tissue-biased expression and subfamily-dependent drought responses, with A, S, I and M subfamilies showing stronger responses to PEG-induced drought stress (**Figure 7**; **Supplementary Tables 18–19**).

Compared with conventional single-reference gene family studies, a pan-genomic framework provides a broader and more informative view of gene family evolution. Previous tea *bZIP* studies mainly identified *bZIP* genes from one reference genome and analyzed their phylogeny, conserved motifs, gene structures, cis-elements and expression patterns; for example, Zhang et al. identified 74 *bZIP* genes in tea plant and classified them into 12 phylogenetic groups, followed by functional characterization of Cs*bZIP*3/42/6 under environmental stresses (Zhang et al., 2024). Although such single-genome studies provide an important foundation for understanding *bZIP* function, they cannot fully capture intraspecific genomic diversity, such as gene presence–absence variation, copy-number variation, cultivar-specific genes and differences between core and dispensable orthogroups. Similar advantages of pan-genomic gene family analysis have recently been demonstrated in barley bHLH, barley NAC, maize GATA and Populus PYL families, where multi-genome approaches uncovered hidden gene members, orthogroup-level variation, relaxed selection in non-core genes and expression divergence associated with structural variation or stress responses (Han et al., 2025; Liu et al., 2025; Tong et al., 2025; Zhao et al., 2025). Therefore, the present study extends tea *bZIP* research from a single-reference identification level to a pan-genomic comparative level, allowing us to distinguish conserved regulatory components from variable members that may contribute to cultivar-specific adaptation and trait divergence.

Transcriptome analysis using ‘Shuchazao’ provided additional evidence for functional differentiation among *bZIP* subfamilies (**Figure 7**). The 81 *bZIP* genes showed broad but tissue-dependent expression across buds, flowers, fruits, leaves, roots and stems (**Figure 7A**; **Supplementary Table 18**). Subfamilies A, B and G were preferentially expressed in fruits, suggesting possible roles in fruit development or reproductive organ regulation (Wang et al., 2023). Subfamily C showed high expression in roots and flowers, while subfamily D was mainly expressed in roots, indicating potential roles in root development or root-related stress responses (Rahman et al., 2024). Subfamily F was broadly expressed across multiple tissues, especially stems, leaves and fruits, suggesting that it may participate in more general developmental or physiological processes (Duan et al., 2022). Core genes showed slightly higher mean expression than dispensable genes in all six tissues, supporting the idea that core *bZIP* members may perform stable and broadly conserved functions in tea plant development. This expression pattern is consistent with pan-genome studies in which core genes are often more broadly expressed and functionally conserved, whereas dispensable genes tend to show more tissue-, genotype- or stress-specific expression patterns (Tao et al., 2025; Sun et al., 2026).

Under PEG-induced drought stress, the tea *bZIP* family displayed clear subfamily-dependent expression responses (**Figure 7B**, **Supplementary Table 19**). Subfamily A showed a gradual induction from 0 h to 72 h, indicating sustained drought responsiveness. This is consistent with the well-established role of A-subfamily *bZIP*s, including ABF/AREB/ABI5-like genes, in ABA-dependent drought and osmotic stress signaling. In blueberry, 102 Vc*bZIP* genes were identified and phylogenetically classified, and the study highlighted their potential value for improving salt and drought stress tolerance (Feng et al., 2025). In soybean, overexpression of Gm*bZIP*60 increased salt and drought tolerance, demonstrating that *bZIP* genes can act as positive regulators of abiotic stress resistance (Chai et al., 2025b). In the present study, subfamilies S and I also showed induced or transient induction patterns under PEG stress, suggesting that they may participate in drought-responsive regulatory networks in tea. Subfamily M showed the highest expression at 72 h, although it was represented by a single gene in the SCZ transcriptome analysis, implying that this member may have a specific function during prolonged drought stress (**Supplementary Table 19**). In contrast, subfamilies C, D and F showed decreased expression after PEG treatment, although F remained highly expressed overall. This suggests that some *bZIP* subfamilies may be downregulated during drought stress to reprogram growth-related processes, while others are activated to enhance stress adaptation. Therefore, drought responses in the tea *bZIP* family are not uniform but are characterized by subfamily-specific activation and repression.

Overall, these findings not only improve our understanding of *bZIP* family expansion and functional divergence in *C. sinensis*, but also provide candidate subfamilies and genes for future studies on drought tolerance, tissue development and molecular breeding of tea plants. In particular, the distinction between core and dispensable *bZIP* genes offers a useful strategy for separating broadly conserved regulatory genes from potentially germplasm-specific adaptive genes, which may facilitate the identification of key transcription factors for tea improvement.

## Data Availability

The original contributions presented in the study are included in the article/**Supplementary Material**. Further inquiries can be directed to the corresponding author.
